# Multi-photon absorption enhancement by dual-wavelength double-pulse laser irradiation for efficient dicing of sapphire wafers

**DOI:** 10.1038/s41598-017-05548-x

**Published:** 2017-07-12

**Authors:** Mindaugas Gedvilas, Justinas Mikšys, Jonas Berzinš, Valdemar Stankevič, Gediminas Račiukaitis

**Affiliations:** Center for Physical Sciences and Technology, Savanoriu Ave. 231, LT-02300 Vilnius, Lithuania

## Abstract

The evidence of multi-photon absorption enhancement by the dual-wavelength double-pulse laser irradiation in transparent sapphire was demonstrated experimentally and explained theoretically for the first time. Two collinearly combined laser beams with the wavelengths of 1064 nm and 355 nm, inter-pulse delay of 0.1 ns, and pulse duration of 10 ps were used to induce intra-volume modifications in sapphire. The theoretical prediction of using a particular orientation angle of 15 degrees of the half-wave plate for the most efficient absorption of laser irradiation is in good agreement with the experimental data. The new innovative effect of multi-photon absorption enhancement by dual-wavelength double-pulse irradiation allowed utilisation of the laser energy up to four times more efficiently for initiation of internal modifications in sapphire. The new absorption enhancement effect has been used for efficient intra-volume dicing and singulation of transparent sapphire wafers. The dicing speed of 150 mm/s was achieved for the 430 μm thick sapphire wafer by using the laser power of 6.8 W at the repetition rate of 100 kHz. This method opens new opportunities for the manufacturers of the GaN-based light-emitting diodes by fast and precise separation of sapphire substrates.

## Introduction

Single crystal sapphire (*α*-Al_2_O_3_) offers superior physical, chemical and optical properties, which make it an excellent material for wide range of applications^1–5^, including high-speed integrated circuit chips; thin-film and GaN-based light-emitting diode substrates^6^; various electronic and mechanical components; wristwatch crystals and movement bearings for the watch industry; scratch resistant display and camera cover for luxury mobile phones; high durability optical components and windows for extreme applications etc. However, sapphire is mechanically and chemically difficult to machine because of its high hardness^7^ and chemical inactivity^8^. Many of the possible applications are restricted because of the high cost of sapphire machining, and it is used only in the expensive devices. Laser processing has been proposed as a potential machining method of sapphire^9–12^. However, the surface laser scribing of sapphire wafers contaminates the substrate. Intra-volume laser dicing of sapphire is a promising technique^13–17^. It allows keeping both top and bottom surfaces absolutely clean and free from ablation debris because the laser modifications are induced in the volume of sapphire. This method also enables to achieve zero-width cut without wasting expensive material. However, sapphire has the wide energy band gap of 9.9 eV and is transparent material in the range of wavelengths from 0.2 μm to 5.5 μm^18^. For the industrial lasers operating at the fundamental wavelength of 1064 nm (photon energy of 1.17 eV), the band gap of sapphire is nine times higher than the photon energy. The extremely high peak intensities are needed to induce the nine-photon absorption (9PA). Therefore, only a small fraction of the laser energy is absorbed in the volume of transparent sapphire by 9PA. The absorbed portion of energy induces internal modifications and cracks. While the rest of the irradiation just goes through the wafer and is wasted making the whole process inefficient. The way to overcome this is to convert laser irradiation to a shorter wavelength by using harmonic generation crystals. Having industrial laser operating at the third harmonics with the wavelength of 355 nm (photon energy of 3.49 eV) the band gap of sapphire is just three times higher than the photon energy. Therefore, only the three-photon absorption (3PA) is needed to induce modifications in the volume of the material. The absorption coefficient of 3PA is several orders of magnitude higher than that of 9PA. Thus, the laser energy can be used more efficiently by using ultraviolet (UV) irradiation instead of the infrared (IR). However, the part of the laser energy and the beam quality are lost in the harmonics generation. That limits the efficient use of the laser energy for the intra-volume dicing of sapphire by using ultra-short UV lasers.

In the last decade, scientific works related to the light-matter interaction utilising the dual-wavelength double-pulse laser irradiation has emerged. The significant enhancement of laser-induced plasma spectroscopy with dual-wavelength femtosecond double-pulse plasma spectroscopy has been reported^19^. The laser-induced periodic surface structure (LIPSS) or ripple formation by the two-colour (UV-IR) double pulse femtosecond laser irradiation on conductors^20, 21^ semiconductors^22^ and dielectrics^23^ has been intensively investigated^24^. The two-colour double-pulse irradiation has been used for laser induced damage thresholds (LIDT) of coatings^25–28^ and has proved to reduce the damage threshold by 71% for Al_2_O_3_ coating^29^. It has been demonstrated that dual-wavelength double pulse laser machining is an efficient way to improve the laser processing of dielectrics by providing a better control of the energy deposition process^30^. The majority of mentioned scientific works related to the dual-wavelength double-pulse irradiation has reduced the damage threshold of transparent material and improved laser energy deposition to the material. The main idea proposed in this work was to use two-colour double-pulse irradiation for efficient dicing of sapphire wafers. Therefore, a small part of the IR laser energy was converted to UV light by the third harmonics generation. The largest part of irradiation was applied to the material in the fundamental IR wavelength of the laser. The UV pulse with the small pulse energy is absorbed via the 1PA or 2PA mechanism and excites electrons from the valence band to the localised states of intrinsic structural defects in sapphire. The later the IR pulse with the large pulse energy is easier absorbed by electrons from the localised state to the conduction band via the 6PA and 3PA. That ensures the more efficient use of the laser energy and enhances the speed of the intra-volume dicing of sapphire wafers.

In this work, the experimental and theoretical results of the efficient volume dicing of sapphire wafers by using the dual-wavelength double-pulse picosecond laser irradiation are presented. The multi-photon absorption enhancement by combined irradiation was demonstrated experimentally and explained theoretically for the first time. The predictions of the theoretical model are in good agreement with the experimental results. The new innovative method allowed usage the energy of the laser irradiation up to four times more efficiently by using the particular ratio between the pulse energies of IR and UV laser pulses. The new technique has been used for efficient intra-volume dicing and singulation of the transparent sapphire wafers.

## Results and Discussion

### Laser-induced intra-volume modifications in sapphire

**Figure 1:**
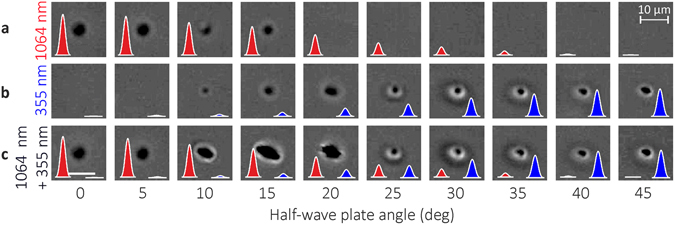
Laser-induced intra-volume modifications in sapphire. Transverse-plane optical microscope images of laser-induced intra-volume modifications in the sapphire wafer. (**a**) Single laser pulse modification at the wavelength of *λ* = 1064 nm; (**b**) single laser pulse modification at the wavelength of *λ* = 355 nm; (**c**) dual-wavelength double-pulse induced modification at wavelengths of *λ* = 1064 nm and *λ* = 355 nm with the delay of Δ*t* ≈ 0.1 ns between pulses (UV prior IR). The inserted red and blue laser pulses graphically represent the decay of IR and growth of UV laser pulse energies with the increase of the orientation angle of the HWP.

Sapphire wafer was irradiated by single laser pulses of IR, by single laser pulses of UV and by the combined double-pulse dual-wavelength irradiation (see Methods section). The pulse energy was varied by changing the orientation angle of the half-wave plate (HWP) (see Methods section). The main purpose of the test was to investigate the energy ratio between the UV and IR laser pulses that induces the largest transverse and longitudinal intra-volume modification sizes of sapphire in the combined irradiation regime. The aim was to find the optimal orientation angle of the HWP for the largest modification size by using the total input pulse energy of 20 μJ of the fundamental IR laser radiation wavelength available from the used laser. The optical images of laser-induced transverse intra-volume modifications in the sapphire wafer by the single pulse and the dual-wavelength double-pulse irradiations are given in Fig. 1.

The size of transverse intra-volume modifications induced by IR irradiation decreases with increasing the orientation angle of HWP (Fig. 1a). It has an inverse relationship for the UV irradiation and increases with increasing HWP angle (Fig. 1b). The size of transverse intra-volume modifications has a clear peak at the HWP angle of ~15 degrees for the combined dual-wavelength double-pulse irradiation (Fig. 1c). The highest total irradiation energy was achieved with the orientation angle of the HWP of *φ* = 0 degrees when none of the IR laser power was converted to the UV. However, the largest transverse-plane modification area in sapphire was obtained at the HWP angle of ~15 degrees (Fig. 1c), when 7% of the IR laser power was converted to the UV. This experimental result suggested that the absorption of the second IR pulse was enhanced by the absorbed first UV pulse in the dual-wavelength double-pulse irradiation.

The shapes of modified areas are elliptical (Fig. 1c, 10–20 deg). The slight misalignment of the collinear UV and IR laser beams in the transverse XY plane resulted in expansion of modified area in one direction and change of modified areas from circular to elliptical. The different contrasts with the bright and dark rings around the dark central spot were observed (Fig. 1c, 25–45 deg). The dark and bright regions correspond to the small negative and positive change of the refractive index of sapphire^31–33^.

### Absorption model of the dual-wavelength double-pulse irradiation

**Figure 2:**
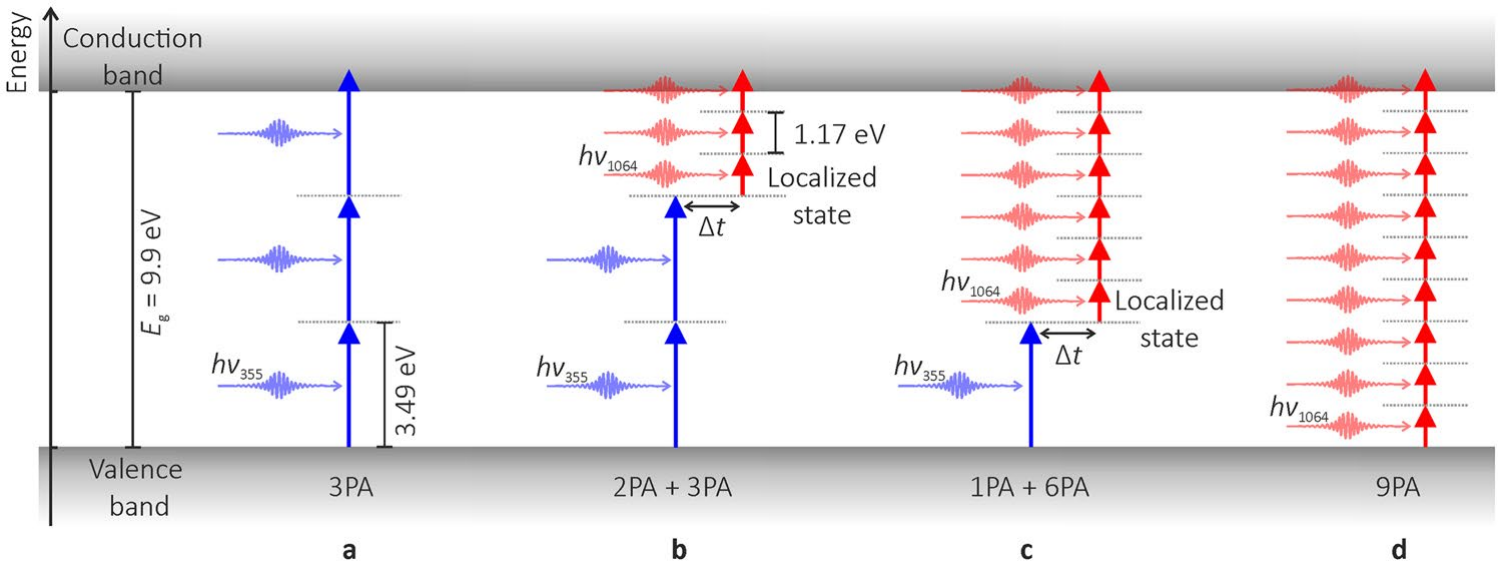
Absorption models of the dual-wavelength double-pulse irradiation. (**a**) 3PA of the UV pulse from the valence to the conduction band; (**b**) 2PA of the UV pulse to a localised state and 3PA of the IR pulse from the localized state to the conduction band; (**c**) 1PA of the UV pulse to a localized state and 6PA of the IR pulse from the localized state to the conduction band; (**d**) 9PA of the IR pulse from the valence to the conduction band.

Four different absorption models are possible in the transparent sapphire (Fig. 2).

In the first absorption model, the UV pulse with a wavelength of 355 nm and the photon energy of 3.49 eV is absorbed via 3PA mechanism driving electrons from the valence band to the conduction band (Fig. 2a). The intensive inter-band excitation by 3PA can facilitate the formation of point defects in the lattice of *α*-Al_2_O_3_. That defects produce localised states in the band-gap at the different energy levels depending on the type of the defect^34^. In the second absorption model, the UV pulse excites electrons from the valence band to the defect-related localised state via 2PA mechanism (Fig. 2b). The absorption band at 7.0 eV is associated with oxygen-Frenkel-pair (${{\rm{V}}}_{{\rm{O}}}^{2+}$ and ${{\rm{O}}}_{{\rm{i}}}^{2-}$) defects in sapphire^18, 35^. The defect related energy level matches properly with the energy of two UV photons of 6.98 eV. The experimentally measured and theoretically calculated lifetimes of the localised state such as the F^+^ colour centre are ~7 ns and ~3.8 ns, respectively^36, 37^. The inter-pulse time delay of Δ*t* ≈ 0.1 ns was selected to be shorter than the relaxation time of electrons in that localised state. Later, after the inter-pulse time delay, the second IR pulse with the wavelength of 1064 nm and the photon energy of 1.17 eV is absorbed and excites the electrons from the localised state to the conduction band via 3PA mechanism (Fig. 2b). In the third absorption model, the first UV pulse is absorbed via 1PA mechanism and drives the electrons from the valence band to the localised state (Fig. 2c). The absorption band at 3.47 eV is associated with the O^2−^ vacancy (${{\rm{V}}}_{{\rm{O}}}^{2-}$) defects in sapphire^18, 35, 38–40^. The defect-related energy level is in agreement with the single UV photon energy of 3.49 eV. The second IR pulse is absorbed via 6PA mechanisms from the localised state to the conduction band (Fig. 2c). In the fourth absorption model, the IR pulse is absorbed via 9PA mechanism moving electrons from the valence band to the conduction band (Fig. 2d). The several values of the inter-pulse delay ranging from −0.4 ns to + 0.8 ns has been tested in this work. The absorption enhancement was observed only for the positive pulse delay when the UV pulse arrives to the material before the IR pulse. However, no significant difference in the size of the modified area was observed for several values of the positive inter-pulse delay in the investigated range.

The complicated calculations of the electron inter-band excitation and relaxation are needed for the simulation of the absorption enhancement^41, 42^. However, instead of analysis complex equation system of electron concentration dynamics in the excited states, the simplified absorption model can be used^42^. In this work, we propose a simple theoretical equation that incorporates all four above mentioned models of absorption for the combined dual-wavelength double-pulse irradiation. Therefore, the absorbed laser power per unit volume can be described as:

1 $$\frac{{\rm{d}}{I}_{355+1064}}{{\rm{d}}z}=-{\alpha }_{3}{I}_{355}^{3}-{\alpha }_{2,3}{I}_{355}^{2}{I}_{1064}^{3}-{\alpha }_{1,6}{I}_{355}{I}_{1064}^{6}-{\alpha }_{9}{I}_{1064}^{9}$$

where *α*_3_ is the 3PA coefficient for the first absorption model (Fig. 2a), *I*_355_ is the peak intensity of the UV pulse, *α*_2,3_ is the total absorption coefficient for the second absorption model (Fig. 2b), *I*_1064_ is the peak intensity of the IR pulse, *α*_1,6_ is the total absorption coefficient for the third absorption model (Fig. 2c), *α*_9_ is the 9PA coefficient for the fourth absorption model (Fig. 2d).

### The theoretical angle of a half-wave plate (HWP) for maximum absorption

**Figure 3:**
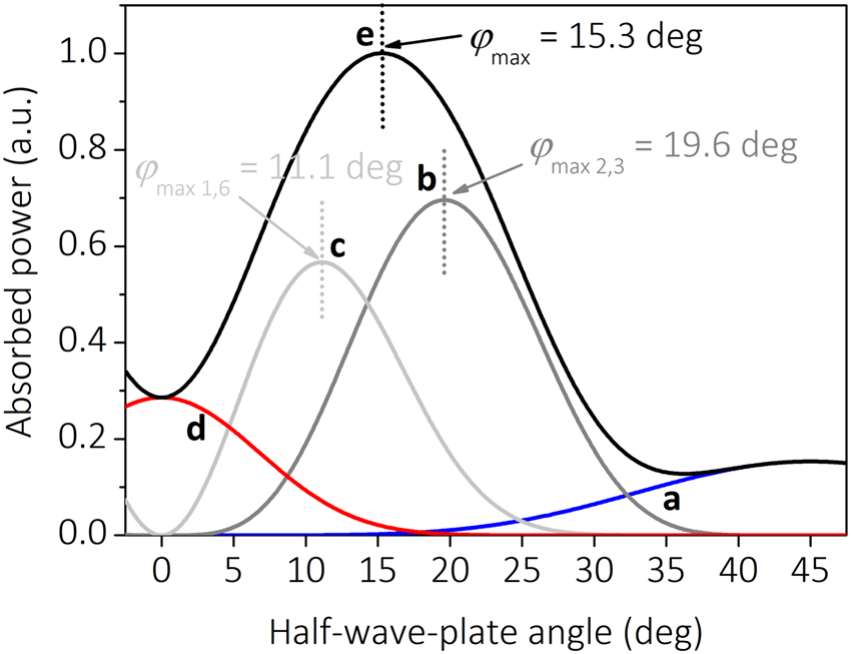
Absorbed laser power versus HWP orientation angle. The calculated absorbed laser power per unit volume versus HWP orientation angle: (**a**) 1^st^ term in Equation (1); (**b**) 2^nd^ term in Equation (1); (**c**) 3^rd^ term in Equation (1); (**d**) 4^th^ term in Equation (1); (**e**) the total absorbed power per unit volume calculated by using entire Equation (1).

The first term on the right side of Equation (1) corresponds to 3PA by the first UV pulse in the double-pulse pair described in Fig. 2a. The second term on the right side of Equation (1) corresponds to 2PA of the UV pulse from the valence band to the localised state and 3PA of the IR pulse from the localised state to the conduction band described in Fig. 2b. The third term on the right side of Equation (1) corresponds to 1PA of the UV pulse from the valence band the localised state and 6PA of the IR pulse from the localised state to the conduction band described in Fig. 2c. The fourth term on the right side of Equation (1) corresponds to 9PA of the second IR pulse in the double-pulse pair described in Fig. 2d. By combining Equations (1), (5) and (6) the absorbed laser power per unit volume can be plotted as a function of the HWP orientation angle (Fig. 3). The absorption coefficients used in the numerical calculations were: *α*_3_ = 0.1 cm^3^/TW^2^, *α*_2,3_ = 0.05 cm^7^/TW^4^, *α*_1,6_ = 1 × 10^−4^ cm^11^/TW^6^ and *α*_9_ = 1 × 10^−7^ cm^15^/TW^8^. The blue curve (Fig. 3a) corresponds to 3PA which is mathematically described by the first term in Equation (1). It has the maximum at the HWP orientation angle of 45 degrees. The grey curve (Fig. 3b) corresponds to for the second absorption model in the dual-wavelength double-pulse irradiation and has a peak at the HWP angle of 19.6 degrees. The optimal HWP orientation angle of the maximum absorbed power per unit volume calculated by using derivatives from Equations (1), (5) and (6) is ${\phi }_{\max 2,3}=0.5\arctan \sqrt{2/3}=19.6\,{\rm{\deg }}$, where “2” - the top number in the fraction corresponds 2PA of the UV pulse, and “3” - the bottom number in the fraction corresponds to the 3PA of the IR pulse. The light grey curve (Fig. 3c) shows the third absorption model in the dual-wavelength double-pulse irradiation and has a peak at the HWP angle of 11.1 degrees.

The optimal HWP orientation angle for the third absorption model analogously calculated by using derivatives from Equations (1), (5) and (6) is ${\phi }_{\max 1,6}=0.5\arctan \sqrt{1/6}=11.1\,{\rm{\deg }}$. Where “1” - the top number in the fraction corresponds to 1PA of the UV pulse, and “6” - the bottom number in the fraction corresponds to 6PA of the IR pulse. The red curve (Fig. 3d) corresponds to 9PA which is mathematically described by the 4^th^ term in Equation (1). It has the maximum at the HWP orientation angle of 0 degrees. The black curve (Fig. 3e) corresponding to the total absorbed laser power per unit volume has a clear peak at the HWP angle of ~15 degrees. It can also be theoretically evaluated by averaging the previously calculated optimal angles of HWP ${\phi }_{\max }=({\phi }_{\max 1,6}+{\phi }_{\max 2,3})/2\approx 15\,{\rm{\deg }}$.

### Experimental verification of the half-wave plate (HWP) angle for maximum absorption

**Figure 4:**
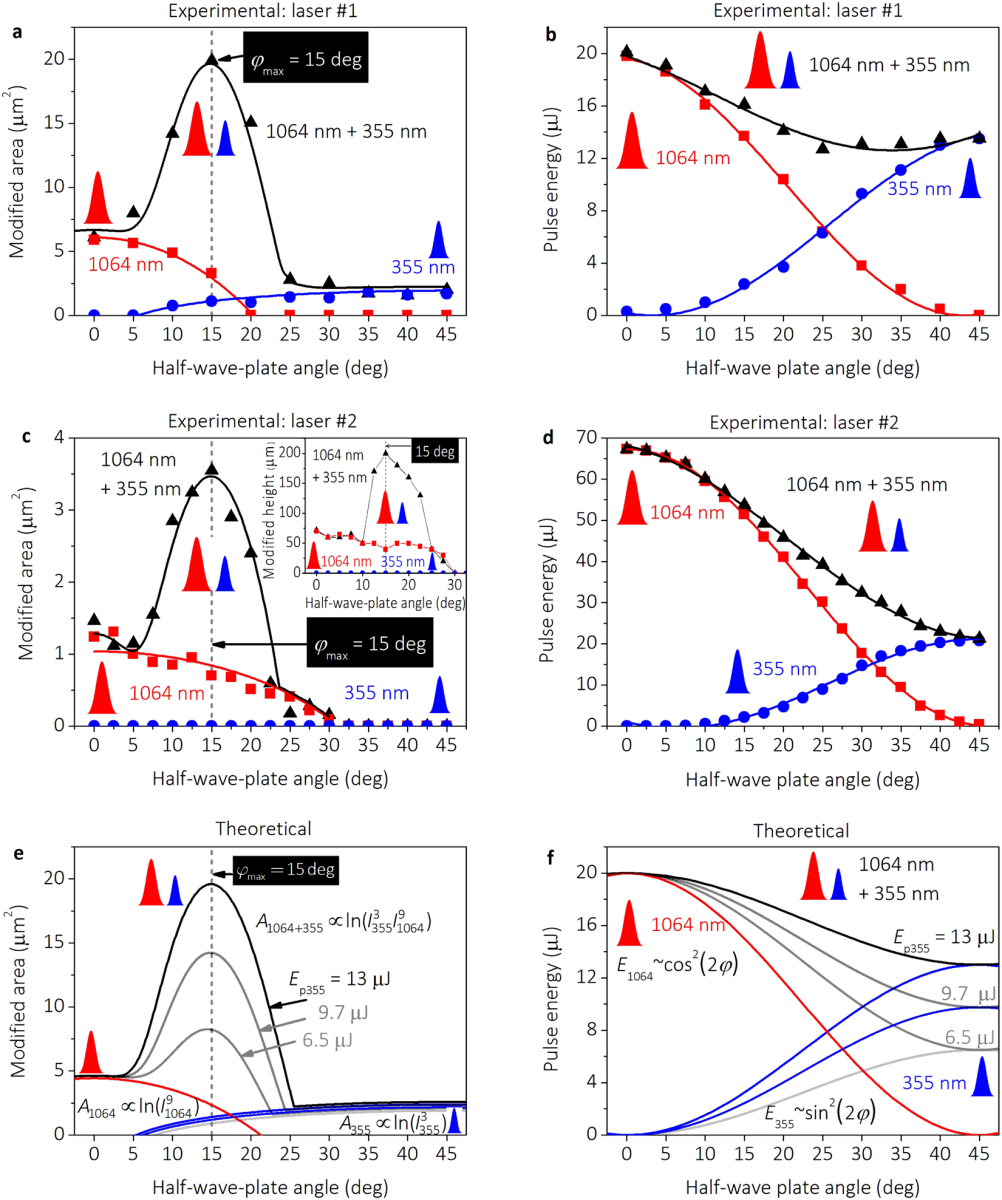
Modification size and pulse energy versus HWP orientation angle. (**a**) Modified area of the intra-volume modification measured in the transverse-plane and (**b**) the pulse energy dependence on the HWP orientation angle: experimental data using the laser source #1. (**c**) Modified area, and (**d**) the pulse energy dependence on the HWP orientation angle: experimental data using the laser source #2. Insert in (**c**) longitudinal modification height dependence on the HWP orientation angle by laser source #2. (**e**) Modified area, and (**f**) pulse energy dependence on the HWP orientation angle: results of the theoretical calculations. Irradiation regimes in (**a**,**b**,**c** and **d**): red solid squares ()– a single IR pulse at the wavelength of *λ* = 1064 nm; blue solid dots ()– a single UV pulse at the wavelength of *λ* = 355 nm; black solid triangles (▲) – the dual-wavelength double-pulse irradiation at the wavelengths of *λ* = 1064 nm and *λ* = 355 nm with the delay Δ*t* ≈ 0.1 ns between pulses in the dual wavelength double-pulse pairs (UV prior IR). Solid lines in all graphs represent results of theoretical modelling.

The dependence of experimentally measured modified area on the HWP orientation angle is given in Fig. 4a,c.

The modified area from the transverse optical microscope images for the single-pulse IR irradiation decreases with the increasing HWP angle (Fig. 4a) because the pulse energy also decreases with the increasing angle of HWP (Fig. 4b). For the single pulse UV irradiation, the modified area increases with the increasing HWP angle (Fig. 4a) because the pulse energy also increases with the increasing angle of HWP (Fig. 4b). However, by using the combined dual-wavelength double-pulse irradiation regime, the modified area has the clear peak with the maximum at the orientation angle of HWP equal to 15 degrees (Fig. 4a). However, the total sum of pulse energies of IR and UV pulses decreases by increasing the HWP angle (Fig. 4b). That is the experimental evidence that combined irradiation of UV and IR pulses can enhance the modification in the transparent sapphire by the factor of ~4. The optimal orientation angle of HWP of 15 degrees is in coincide well with the theoretical prediction obtained by calculating the maximum absorbed laser power using Equation (1) (Fig. 3e). The promising results obtained by using laser irradiation source #1 with the low average power and repetition rate inspired us to use more powerful and high repetition rate laser #2 in order to upscale the observed effect. The conversion efficiency to the third harmonics was less for the laser #2 (Fig. 4d) than for the laser #1 (Fig. 4b). Moreover, the spot sizes of collinearly combined beams were increased by factor of 2 in order to have larger Rayleigh lengths and its related modification heights (see Methods section Experimental set-up). Therefore, the modification using a single-pulse irradiation by the UV pulse was not observed (Fig. 4c). The modification areas versus the HWP angle for the single pulse IR irradiation and the dual-wavelength double-pulse combined irradiation using the laser #2 (Fig. 4c) had similar dependence as in the case of the laser #1 (Fig. 4a). The size of modified area also had a clear peak with the maximum position at the orientation angle of the HWP of 15 degrees (Fig. 4c). The similar peak was observed in the longitudinal modification height measured by using an optical microscope (insert in Fig. 4c). The enhancement of transverse modified area and the longitudinal modification height by the factor of ~4 was experimentally recorded for two different laser irradiation sources #1 and #2. The theoretical modelling was employed in order to explain the experimental results. The theoretically calculated transverse modification area versus the HWP angle is given in (Fig. 4e). The theoretically calculated modification area had a distinctive peak with the maximum at the HWP angle of 15 degrees. The peak position of the absorbed power per unit volume did not depend on the conversion efficiency of the IR irradiation to the UV by the harmonics crystal and had the same position of ~15 degrees (Fig. 4e). However, the sum of pulse energies in the dual-wavelength double-pulse regime was always smaller than the pulse energy of the IR irradiation at the orientation angle of HWP of 0 degrees (Fig. 4f). The theoretical prediction that the most efficient absorption in the dual-wavelength double-pulse picosecond laser irradiation occurs with the ~15 degrees orientation angle of the HWP is in good agreement with the experimental results.

### Efficient intra-volume dicing of the sapphire wafer by combined laser irradiation

**Figure 5:**
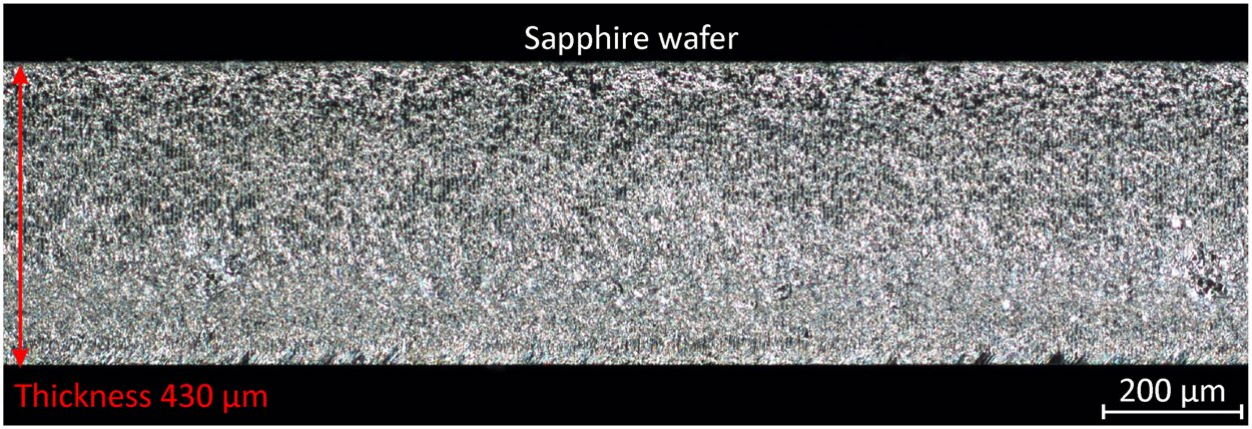
Image of a sapphire sidewall after laser dicing. The dark field optical microscope image of the fracture plane containing subsurface modifications induced by the dual-wavelength double-pulse laser irradiation. Four layers of merged modifications at different focus depths formed a uniform fracture plane. The lateral spacing between the laser-induced modifications was 6 μm. Processing parameters: HWP orientation angle *φ* = 15 degrees; UV pulse energy *E*_p355_ = 2.2 μJ; IR pulse energy *E*_p1064_ = 51.5 μJ; scanning speed 600 mm/s; laser pulse repetition rate 100 kHz; pulse duration 10 ps; time delay Δ*t* ≈ 0.1 ns between pulses (UV prior IR); number of scans 4; focal position difference in each of the scan 90 μm; total dicing speed 150 mm/s.

The unique effect of the absorption enhancement was applied for the efficient intra-volume dicing of sapphire. The combined dual-wavelength double-pulsed picosecond laser beam was focused in the volume of the sapphire wafer with the thickness of 430 µm. The sample was moved at the speed of 600 mm/s by using a linear translation stage. The internal modifications were initiated in the volume of sapphire. Four scans were performed at different depths of sapphire by moving up the position of focusing objective by 90 μm. A small force was applied to the wafer, and its singulation along the direction of the laser induced modifications was achieved (Fig. 5).

The total dicing speed of 150 mm/s was achieved. The modifications were induced in the volume of sapphire keeping the top and bottom surfaces undamaged.

## Conclusions

It was demonstrated for the first time experimentally and confirmed theoretically that multi-photon absorption could be enhanced by the combined dual-wavelength double-pulse irradiation in the transparent sapphire. The absorption enhancement has been used for efficient intra-volume dicing of the sapphire wafer by using the combined picosecond laser irradiation. The transverse modification size in the volume of the transparent sapphire was increased by the factor of four by using the particular orientation angle of the half-wave plate of ~15 degrees also predicted theoretically by the analytical expression. The successful laser dicing of the sapphire wafer with the thickness of 430 μm was achieved by using the dual-wavelength double-pulse laser irradiation. The total dicing speed of 150 mm/s was reached by using laser irradiation source with the power of 6.8 W, the pulse duration of 10 ps and the repetition rate of 100 kHz. This new technique opens possibilities for the fast cutting of sapphire substrates, and it is important for the manufacturers of the GaN-based light-emitting diodes. The innovative results are promising for the future applications of the efficient use of laser energy in machining of sapphire and other transparent materials.

## Methods

### Material

The synthetic single crystal sapphire (*α*-Al_2_O_3_, CrystalQ) was used in the experiments. The parameters of the sapphire wafer: orientation C-plane (0001); off-cut 0.30 deg to M-plane; primary flat orientation A-plane; diameter 50.8 mm; thickness 430 μm; finish - double sided epi-polished; top surface roughness of Ra ≤ 0.3 nm; bottom surface roughness Ra ≤ 0.5 nm.

### Experimental set-up

**Figure 6:**
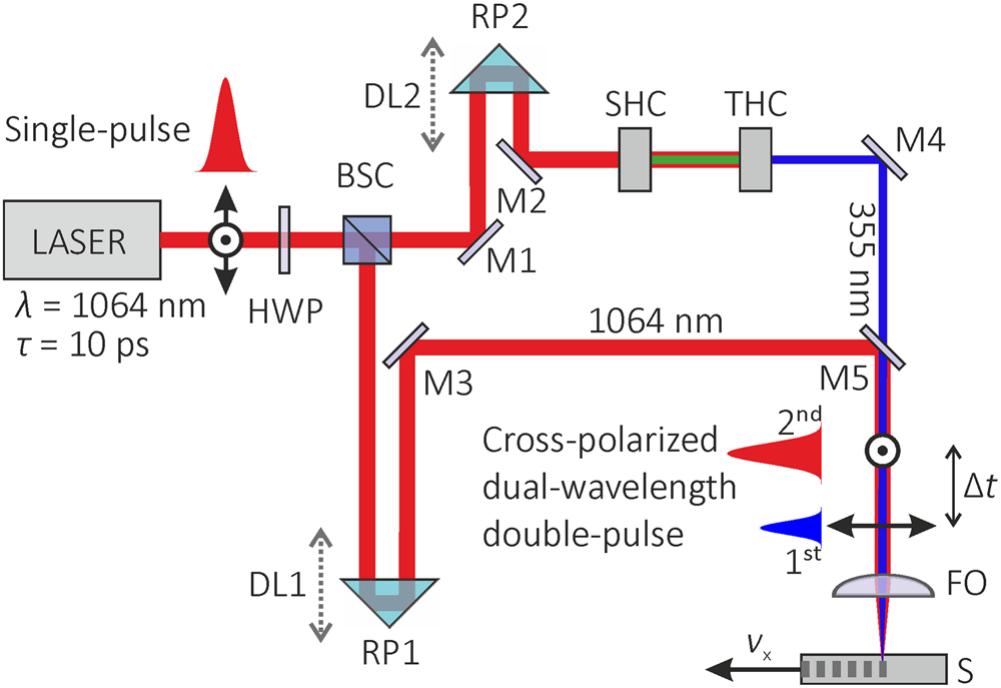
Experimental setup for the cross-polarized dual-wavelength double-pulse combined laser irradiation. LASER is the pulsed laser radiation source; HWP is the half-wave plate; BSC is the beam splitter cube; DL1 and DL2 are the delay lines with movable retro-reflection prisms RP1 and RP2; M1, M2, M3, and M4 are the high reflective mirrors; SHC is the second harmonic crystal; THC is the third harmonic crystal; M5 is the harmonic beam splitter mirror; FO is the focusing objective, S is a sapphire wafer sample, *v*_x_ is the scanning speed of sapphire sample.

The principal scheme of the experimental setup for the cross-polarized dual-wavelength double-pulse combined irradiation is presented in Fig. 6.

In the experimental setup, laser light was separated into two beams by the beam splitter cube. The bottom beam path was designed for IR irradiation at the wavelength of 1064 nm. The UV irradiation at the wavelength of 355 nm was generated in the top beam path by using the second and third harmonic crystals. For generation of the second harmonics, LBO type-I nonlinear crystal with the size of 5 × 5 × 10 mm^3^ was used. For generation of the third harmonics, LBO type-II nonlinear crystal with the size of 5 × 5 × 8 mm^3^ was used. Both beams were collinearly combined by using the harmonic beam splitter mirror. The combined cross-polarized dual-wavelength double-pulsed beam was focused in the volume of the sapphire wafer by using the aspheric uncoated focusing objective with the focal length of 8.0 mm and the numerical aperture of 0.50 (A240TM, Thorlabs). The time delay between laser pulses in the double-pulse pairs was controlled by optical delay lines with retro-reflector prisms and was set to Δ*t* ≈ 0.1 ns. The ratio between laser pulse energies of IR and UV pulses was controlled by the orientation angle of the HWP before the beam splitter cube. The sapphire wafer was moved at certain speeds providing the controllable distance between the transverse beams spots focused in the volume of sapphire wafer. Two industrial-grade diode-pumped pulsed lasers (PL10100, Ekspla) and (Atlantic, Ekpsla) with the pulse durations of *τ* = 10 ps irradiation in the IR region at the wavelength of *λ* = 1064 nm were used in the experiments. The first laser provided laser pulses with the pulse energy up to 20 μJ at a repetition rate of *f*_rep_ = 1 kHz with the average laser power of 0.020 W. The laser spot sizes of the focused Gaussian beams in the volume of sapphire were measured by the technique described by Liu^43^. For the first laser source, the spot size radiuses were *w*_0 355_ ≈ 3 μm and *w*_0 1064_ ≈ 3 μm with the Rayleigh lengths of *z*_R 355_ ≈ 80 μm and *z*_R 1064_ ≈ 27 μm of third and fundamental harmonics, respectively. The second laser with the same laser pulse duration and irradiation wavelength but higher repetition rate and pulse energy was chosen in order to upscale the modification effect achieved with the first low repetition rate laser. The second laser source provided radiation with the pulse energy of 68 μJ at the repetition rate of *f*_rep_ = 100 kHz with the average power of 6.8 W. The spot sizes for the second laser source were decreased in order to have larger Rayleigh lengths and with the related higher longitudinal modification depth. The measured the spot size radiuses were *w*_0 355_ ≈ 6 μm and *w*_0 1064_ ≈ 6 μm with the Rayleigh lengths of *z*_R 355_ ≈ 318 μm and *z*_R 1064_ ≈ 106 μm of third and fundamental harmonics, respectively.

### Irradiation regimes

**Figure 7:**
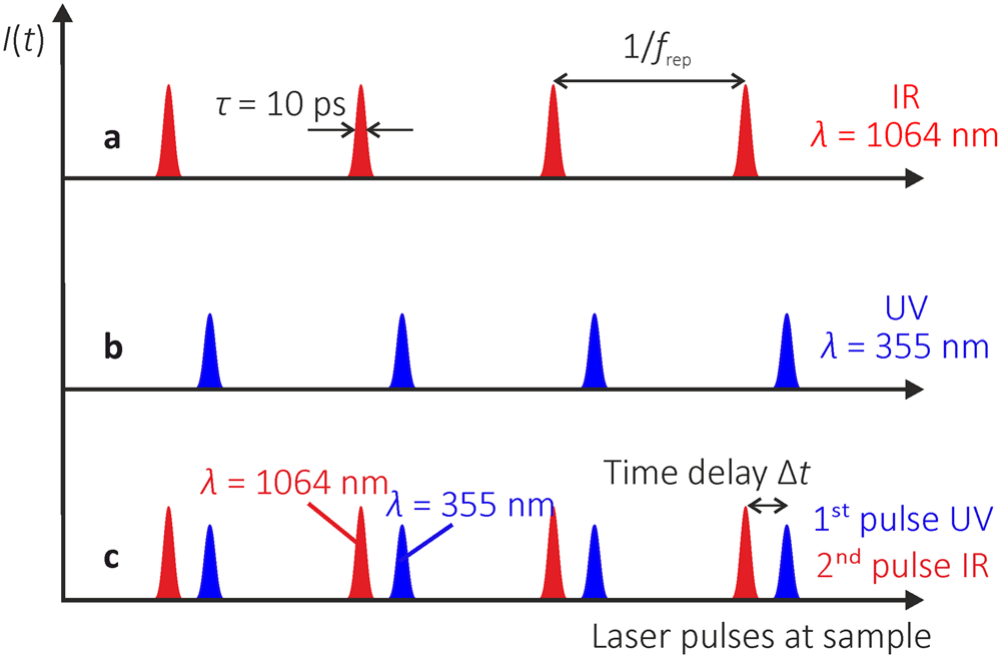
Principal scheme of the irradiation regimes. Schematic representation of the laser intensity versus arrival time of laser pulses at the sample: (**a**) single-pulse IR irradiation at the wavelength of 1064 nm; (**b**) single-pulse UV irradiation at the wavelength of 355 nm; (**c**) dual-wavelength double-pulse combined irradiation with the variable inter-pulse delay.

Two regimes of irradiation were used in the experiments: the single-pulse irradiation (Fig. 7a,b) and dual-wavelength double-pulse combined irradiation (Fig. 7c).

The single-pulse train irradiation was used by using two laser wavelengths: either *λ* = 1064 nm (Fig. 7a) or *λ* = 355 nm (Fig. 7b). In the dual-wavelength double-pulse irradiation scheme, the dual-wavelength (*λ* = 1064 nm and *λ* = 355 nm) pulse pairs with the delay Δ*t* between them were used for irradiation of the samples. The delay Δ*t* ≈ 0.1 ns represents the situation when UV pulse reaches the sapphire sample first in the double-pulse pair, and the IR pulse is the second one (Fig. 7c). The temporal distance between repetitive laser pulses or double-pulse pairs was 1/*f*_rep_, where *f*_rep_ is the laser repetition rate.

### Characterization of laser induced modifications in sapphire

The transverse and longitudinal internal modifications of sapphire were investigated by using an optical microscope (Eclipse LV100, Nikon) equipped with the high-definition 5-megapixel CCD camera (DS-Fi1, Nikon) with the resolution of 2560 × 920 pixels. The digital camera was controlled by the microscope camera controller (Digital Sight DS-U2, Nikon) and the imaging software (NIS-Elements D, Nikon). The microscope objective (LU Plan Fluor 50×, Nikon) with the magnification factor of 50× and the numerical aperture of 0.8 was used in the dark field mode. The illumination source of the microscope consisted of a 50 W halogen lamp (LV-HL50PC, Nikon). The free and open source software (Gwyddion, Czech Metrology Institute) was used for image analysis and evaluation of the laser modified areas in the volume of sapphire.

### Modification size in multi-photon absorption (MPA)

The absorbed laser power per unit volume for the MPA^44^:

2 $$\frac{{\rm{d}}I}{{\rm{d}}z}=-{\alpha }_{N}{I}^{N},$$

where *I* is the peak laser intensity, *z* is the direction along beam propagation axis, *α*_*N*_ is the MPA coefficient, *N* is the number of the photons needed to overcome the energy band gap in the transparent material. The transverse modification area *A* in the MPA^45^:

3 $$A=\frac{2\pi {w}_{0}^{2}}{N}\,\mathrm{ln}(\frac{I}{{I}_{{\rm{th}}}}),$$

where *w*_0_ is the Gaussian beam radius, *I*_th_ is the modification threshold. The longitudinal modification height can be defined as follows^45^:

4 $$h=2{z}_{{\rm{R}}}\sqrt{{(\frac{I}{{I}_{{\rm{th}}}})}^{\frac{1}{N}}-1},$$

where *z*_R_ is the Rayleigh length of the Gaussian beam. The intensity threshold for sapphire modification *I*_th_ = 10^13^ W/cm^2^ was used in the numerical calculations^46, 47^.

### Peak laser intensity versus orientation angle of a half-wave plate (HWP)

The peak laser intensity of the IR irradiation is proportional to the pulse energy and decreases with increasing HWP orientation angle by the cosine law (Fig. 4b,d,f):

5 $${I}_{1064}(\phi )\propto {E}_{1064}(\phi )={E}_{1064}^{0}{\cos }^{2}(2\phi ),$$

where *φ* - is the angle of HWP orientation with the reference (zero) orientation angle, corresponding to the whole energy directed to the IR optical beam path, *E*°_1064_ = 20 μJ and *E* ^0^_1064_ = 68 μJ - is the maximum pulse energy of the fundamental harmonics of the first and second laser sources, respectively. The peak laser intensity of UV irradiation is proportional to the pulse energy and increases with the orientation angle of the HWP by sinus function (Fig. 4b,d,f):

6 $${I}_{355}(\phi )\propto {E}_{355}(\phi )={E}_{355}^{0}\,{\sin }^{2}(2\phi ),$$

where *E* ^0^_355_ = 14 μJ and *E* ^0^_355_ = 21 μJ - is the maximum pulse energy of the third harmonics of the first and second laser sources, respectively. Equations (1) and (2) defining the absorbed energy for all three irradiation regimes were combined with Equations (3) and (4) defining the size on the transverse and longitudinal sizes of the modified regions. The IR and UV pulse energy dependence on the orientation angle of the HWP taken from Equations (5) and (6). All Equations were combined and numerically solved by using (Maple, Waterloo Maple) computing software.
